# Adjunct pharmacotherapy for psychotherapy

**DOI:** 10.1192/bjo.2021.460

**Published:** 2021-06-18

**Authors:** Jessica Lochtenberg, Ari Kirshenbaum, Matthew Johnson

**Affiliations:** 1 Royal College of Surgeons in Ireland; 2 Saint Michael's College; 3 Johns Hopkins University

## Abstract

**Aims:**

A variety of pharmacotherapies have been used to assist the psychotherapy process as “adjunctive therapies.” These drugs are used in an acute, targeted fashion, such that they are explicitly delivered in the context of psychotherapy for anxiety, mood and substance-dependence disorders (SUDs). Our narrative review highlights the potential of medically-assisted psychotherapy by outlining the current state of research on few of these medications and describing the basic science that supports their use.

**Method:**

Firstly, we researched an assortment of medications that have been used off-label to enhance psychotherapy, and selected a few that have received the most empirical attention in preclinical and clinical-trial settings. Our review of clinical trials focused on three of the most common psychiatric ailments. For all studies reviewed, we identify the strengths and weaknesses of the data supporting the use of the medications for the three aforementioned disorders.

**Result:**

D-cycloserine: accelerates the process of associative emotional learning, enhancing exposure therapy in the treatment of various anxiety disorders, including obsessive-compulsive disorder and posttraumatic stress disorder. Limited studies are available on efficacy in treating SUDs.

Intranasal oxytocin: accelerates memory retrieval-extinction procedures used in posttraumatic stress disorder, and promotes prosocial cognition and behaviour, facilitating a therapeutic alliance. Sufficiently powered studies and safety studies are required before strong conclusions can be made.

Propranolol: interrupts the reconsolidation of memories (leading to maladaptive learned responses) involved in posttraumatic stress disorder during memory-reactivation therapy sessions, but there is little evidence that this drug can be used for depression or SUDs.

Psychedelics: may effect the brain's default mode network, engendering a transformative experience that is often followed by a reduction in psychiatric symptoms. 3,4-methylenedioxymethamphetamine may additionally modulate the amygdala response in a way that allows for reprocessing of traumatic memories, and improves the therapeutic alliance. Anxiety, mood, and SUDs appear to be positively influence by traditional and non-traditional (ketamine) psychedelics.

**Conclusion:**

Although the efficacy of the medically-assisted psychotherapies reviewed is still under investigation, we propose that these novel treatment approaches may be preferred over traditional psychopharmacological treatments due to the presence of fewer chronic side effects, as well less toxicity and abuse potential. Furthermore, these adjunctive pharmacotherapies may help to reinforce the psychotherapeutic alliance and may ultimately yield better long-term treatment outcomes. If at least some of the adjunctive pharmacotherapies outlined in this review are found to be clinically efficacious and safe, patients will benefit from having more treatment options available to them in the future.

